# Breastfeeding Duration and Airway Inflammation in Children With Asthma Living in a Polluted Megacity

**DOI:** 10.1111/crj.70172

**Published:** 2026-02-15

**Authors:** Víctor González‐Uribe, Zaira Selene Mojica‐González, Jimena Prieto‐Gómez, Ricardo Martinez‐Tenopala, Tamara Hernández‐Hernández, María Julia Rendón‐Salazar, Luis Ángel Hernández‐Zárate

**Affiliations:** ^1^ AlergiaMx Mexico City Mexico; ^2^ Facultad Mexicana de Medicina Universidad La Salle Mexico City Mexico; ^3^ Hospital General de México “Dr. Eduardo Liceaga” Mexico City Mexico; ^4^ Hospital Infantil de México Federico Gómez Mexico City Mexico

**Keywords:** air pollution, asthma, breastfeeding, environmental exposure, FeNO, pediatric allergy

## Abstract

**Background:**

Children with asthma living in highly polluted megacities are at increased risk of poor asthma control and airway inflammation. Breastfeeding has immunomodulatory effects, but its potential to buffer pollution‐related inflammation remains understudied.

**Objective:**

To evaluate whether breastfeeding duration is associated with asthma control and airway eosinophilic inflammation (FeNO), and whether it modifies the impact of urban air pollution in children with asthma living in Mexico City.

**Methods:**

We conducted a multicenter retrospective study (2022–2025) including children aged 6–17 years with physician‐diagnosed asthma. Asthma control (ACT), airway inflammation (FeNO, NIOX VERO), breastfeeding duration (0–12 months), and borough‐level pollution categories (high/medium/low) were evaluated. Multivariable logistic and linear regression models assessed associations and breastfeeding × pollution interactions.

**Results:**

A total of 162 children were included (mean age 10.8 years; 51.2% female). Breastfeeding ≥ 6 months was associated with better asthma control (ACT ≥ 20: OR 2.1; 95% CI 1.3–3.5) and significantly lower FeNO (β −0.24; *p* < 0.01). High‐pollution residence was linked to lower ACT scores and higher FeNO (both *p* < 0.01). A significant interaction indicated that breastfeeding ≥ 6 months attenuated the detrimental effect of pollution on airway inflammation and asthma control (*p* for interaction < 0.05).

**Conclusions:**

Prolonged breastfeeding was independently associated with improved asthma control and reduced airway eosinophilic inflammation, partially mitigating the respiratory burden of chronic pollution exposure.

**Clinical Implications:**

Breastfeeding may serve as an accessible, low‐cost protective strategy for children with asthma living in polluted urban environments, supporting its integration into respiratory health policies and early‐life preventive care.

## Clinical Relevance

- Children with asthma exposed to high levels of urban pollution show increased airway inflammation.
- This study identifies breastfeeding ≥ 6 months as a protective factor that attenuates pollution‐related increases in FeNO and improves asthma control.
- These findings support implementing breastfeeding promotion as part of integrated pediatric asthma care in megacities.

## Graphical abstract

Prolonged breastfeeding (≥ 6 months) was associated with better asthma control and lower airway inflammation (FeNO) in children living in polluted areas of Mexico City. These findings suggest that breastfeeding provides a protective effect against the respiratory consequences of urban air pollution in pediatric asthma.

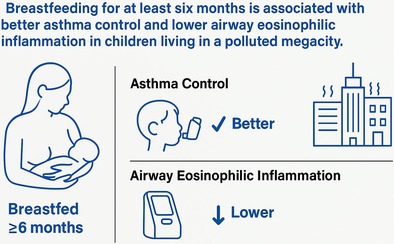

## 1 Introduction

Childhood asthma remains one of the most prevalent chronic conditions worldwide and is shaped by the interplay of genetic susceptibility, early‐life factors, and environmental exposures—particularly urban air pollution [1, 2, 3, 4, 5]. In megacities such as Mexico City, ozone (O_3_), nitrogen dioxide (NO_2_), and fine particulate matter (PM_2.5_) have been repeatedly linked to exacerbations and healthcare utilization in children [2, 6, 7, 8]. Contemporary strategy documents underscore that even when symptom control appears adequate, environmental triggers can precipitate poor outcomes [8, 9, 10, 11].

Breastfeeding has long been hypothesized to reduce asthma morbidity through immunomodulatory pathways, and recent evidence continues to support its role in childhood respiratory health (e.g., secretory IgA, human milk oligosaccharides, and shaping of the infant microbiome) [2, 6, 12, 13]. Recent syntheses continue to support an inverse association between longer breastfeeding and asthma‐related outcomes in childhood, with pooled estimates suggesting modest but meaningful risk reductions as duration increases. Emerging studies from 2022 to 2025 further support these associations [14, 15].

Fractional exhaled nitric oxide (FeNO) is a noninvasive biomarker of type‐2/eosinophilic airway inflammation that correlates with poor control and corticosteroid responsiveness [1, 16, 17, 18]. Current guidance (ATS/ERS, GINA 2024) integrates FeNO into pediatric assessment, with widely used thresholds around 35 ppb and pragmatic cut‐points up to 50 ppb depending on context [1, 17, 18, 19].

In Mexico City, both historical and contemporary evidence links daily and seasonal variation in pollutants with pediatric morbidity [7, 10, 11, 20, 21, 22, 23]. Recent analyses continue to show short‐term pollution spikes associated with asthma exacerbations [22, 24, 25]. Despite regulatory progress, air quality remains a challenge [21, 25].

Against this background, breastfeeding could plausibly buffer the adverse respiratory effects of chronic urban pollution through anti‐inflammatory and barrier‐supporting mechanisms. Yet pediatric studies testing effect modification remain scarce. Building on updated asthma management guidance and contemporary epidemiology in Mexico City, the present study evaluates the association between breastfeeding duration, asthma control (ACT), and airway eosinophilic inflammation (FeNO), and tests whether breastfeeding modifies the pollution–asthma relationship.

## 2 Materials and Methods

This multicenter retrospective observational study was conducted from January 2022 to January 2025 in three pediatric allergy and immunology centers in Mexico City. Children aged 6 to 17 years with a physician‐confirmed diagnosis of asthma, based on GINA 2024 criteria, were eligible. To ensure consistent environmental exposure, only those who had resided in the same borough for at least two consecutive years were included. Exclusion criteria comprised severe exacerbations requiring hospitalization, chronic lung disease, immunodeficiency, and systemic corticosteroid use within 4 weeks before evaluation. All clinical data were extracted from electronic and paper‐based medical records.

Asthma control was assessed using the Asthma Control Test [26], with scores of 20 or higher interpreted as adequate control [26, 27]. FeNO levels were measured using the NIOX VERO device, following the standards of the American Thoracic Society and the European Respiratory Society [17, 18], and values were expressed in parts per billion. Breastfeeding duration was obtained from medical records and parental reports and categorized into 0, 3, 6, 9, or 12 months. Due to the retrospective design, breastfeeding duration reflects cumulative months of any breastfeeding, as recorded in medical charts and parental reports; data on exclusivity, daily frequency, or number of feedings per day were not consistently available. Additional variables included age, sex, body mass index, family history of asthma, and current controller treatment.

Environmental exposure was estimated using data from the Mexico City Atmospheric Monitoring System (SIMAT). Boroughs were classified into high, medium, or low pollution categories based on long‐term ozone IMECA patterns (2019–2023) [7, 21], consistent with previous surveillance reports. Boroughs with frequent exceedances of ozone regulatory standards—Álvaro Obregón, Benito Juárez, Coyoacán, Magdalena Contreras, Miguel Hidalgo, Tlalpan, and Xochimilco—were categorized as high‐pollution areas. Cuauhtémoc, Gustavo A. Madero, Iztapalapa, and Venustiano Carranza were considered medium‐pollution boroughs, while Azcapotzalco, Iztacalco, Milpa Alta, and Tláhuac were classified as low‐pollution areas. This stratification enabled a more precise evaluation of the interaction between breastfeeding duration and pollution burden on asthma outcomes.

Descriptive statistics summarized demographic and clinical characteristics. Continuous variables were compared using *t*‐tests or Kruskal–Wallis tests, and categorical variables using χ^2^ tests. Multivariable logistic regression models evaluated the association between breastfeeding duration and adequate asthma control (ACT ≥ 20), while linear regression models assessed predictors of log‐transformed FeNO. Both models incorporated breastfeeding duration, pollution category, and an interaction term (breastfeeding × pollution), with adjustment for age, sex, and BMI. Missing data accounted for less than 5% of all variables and were handled using complete‐case analysis because the pattern of missingness was random and did not influence model stability. Statistical analyses were performed using R version 4.3.2, and a two‐tailed *p*‐value < 0.05 was considered significant.

The study adhered to the principles of the Declaration of Helsinki and the International Committee of Medical Journal Editors (ICMJE). The protocol was approved by the institutional ethics committees of all participating centers. Given the retrospective design and use of anonymized records, the requirement for informed consent was waived, and patient confidentiality was maintained throughout.

## 3 Results

A total of 162 children were included, with a mean age of 10.8 ± 3.1 years and a balanced sex distribution (51.2% female). Table 1 summarizes the demographic and clinical characteristics of the study population according to breastfeeding duration and borough‐level pollution exposure [7]. Approximately 12% of participants had no breastfeeding history, 39% were breastfed for 3 months, 30% for 6 months, 13% for 9 months, and 6% for 12 months. Most patients resided in boroughs classified as high or medium pollution, reflecting the demographic distribution of the metropolitan area [7].

**TABLE 1:** Demographic and clinical characteristics of the study population.

Variable	Total (*N* = 162)	Breastfeeding < 6 months (*n* = 86)	Breastfeeding ≥ 6 months (*n* = 76)	*p*
Age, years (mean ± SD)	10.8 ± 3.1	10.6 ± 3.0	11.0 ± 3.2	0.42
Sex, female (%)	83 (51.2)	46 (53.5)	37 (48.7)	0.56
BMI, kg/m^2^ (mean ± SD)	19.7 ± 3.2	19.9 ± 3.1	19.4 ± 3.4	0.38
Family history of asthma (%)	94 (58.0)	54 (62.8)	40 (52.6)	0.19
Borough pollution category—high (%)	60 (37.0)	38 (44.2)	22 (28.9)	< 0.01
Borough pollution category—medium (%)	67 (41.4)	33 (38.4)	34 (44.7)	N/A
Borough pollution category—low (%)	35 (21.6)	15 (17.4)	20 (26.3)	N/A
Asthma control (ACT ≥ 20) (%)	110 (67.9)	53 (61.6)	62 (81.6)	0.004
FeNO, ppb (median [IQR])	27 [18–39]	34 [25–46]	21 [15–29]	0.002
Current controller therapy—ICS alone (%)	52 (32.1)	28 (32.6)	24 (31.6)	0.41
Current controller therapy—ICS/LABA (%)	70 (43.2)	35 (40.7)	35 (46.1)	N/A
Current controller therapy—ICS/LABA + LTRA (%)	40 (24.7)	23 (26.7)	17 (22.4)	N/A

*Note:* Demographic and clinical characteristics of the study population (*N* = 162). Data are presented as mean ± standard deviation (SD) for normally distributed continuous variables, median (interquartile range, IQR) for non‐normally distributed continuous variables, and number (percentage) for categorical variables. *p*‐values correspond to χ^2^ tests for categorical comparisons and *t*‐test or Mann–Whitney *U* test for continuous variables, as appropriate. For variables with multiple categorical levels or insufficient cell sizes, statistical comparison was not performed; therefore, *p*‐values are reported as N/A.

Abbreviations: ACT = Asthma Control Test; BMI = body mass index; FeNO = fractional exhaled nitric oxide; ICS = inhaled corticosteroid; LABA = long‐acting β_2_‐agonist; LTRA = leukotriene receptor antagonist.

**FIGURE 1:**
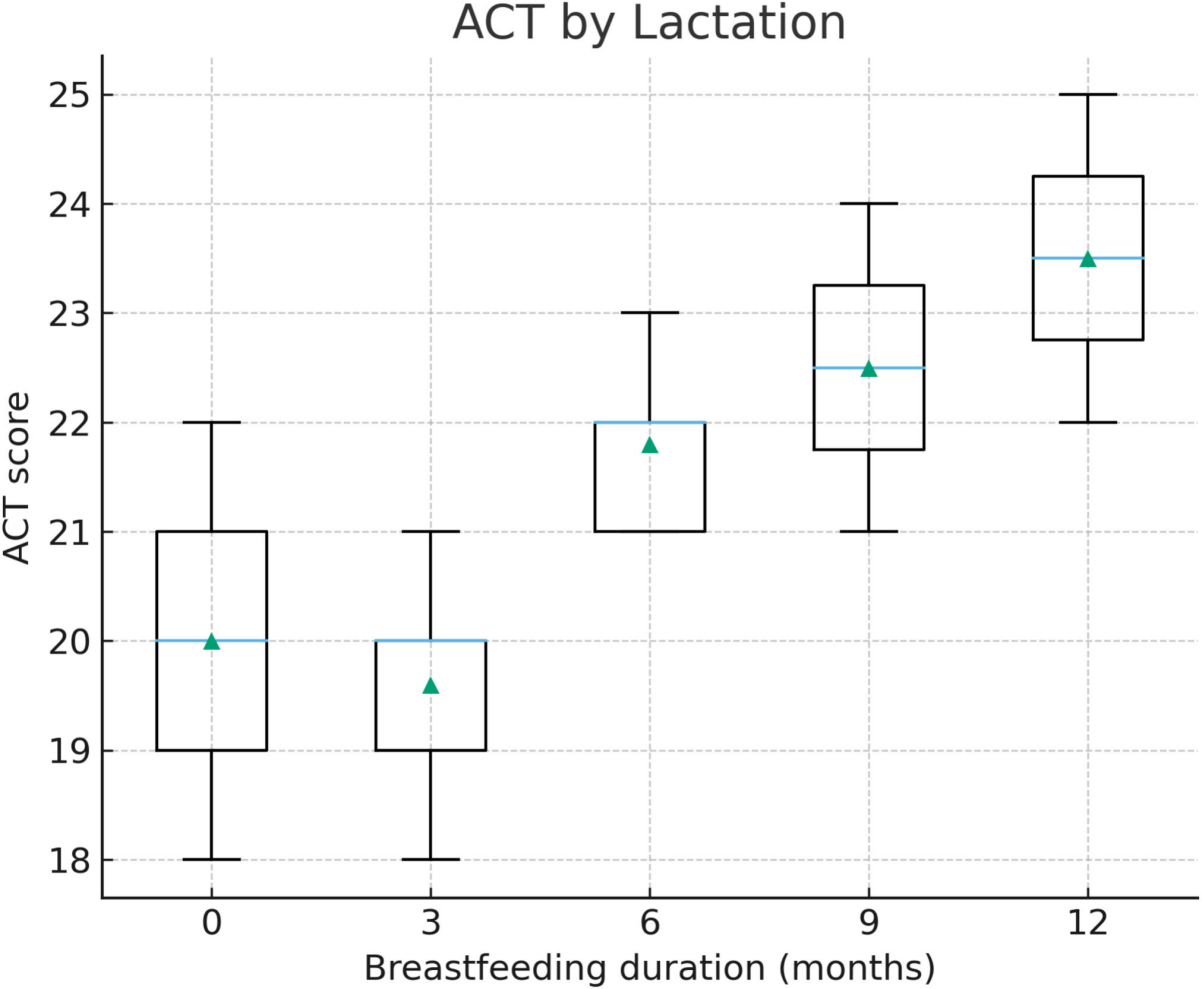
Asthma Control Test (ACT) scores by breastfeeding duration. Boxplots display median, interquartile range, and outliers of ACT scores stratified by breastfeeding duration (0, 3, 6, 9, or 12 months). Group sizes were as follows: < 6 months breastfeeding (*n* = 86) and ≥ 6 months breastfeeding (*n* = 76). Differences were statistically significant (*p* < 0.01). Children breastfed for ≥ 6 months showed significantly higher ACT scores compared with those breastfed for shorter durations or none (*p* < 0.01). Boxes represent IQR, horizontal lines depict medians, and whiskers denote the 10th–90th percentiles.

**FIGURE 2:**
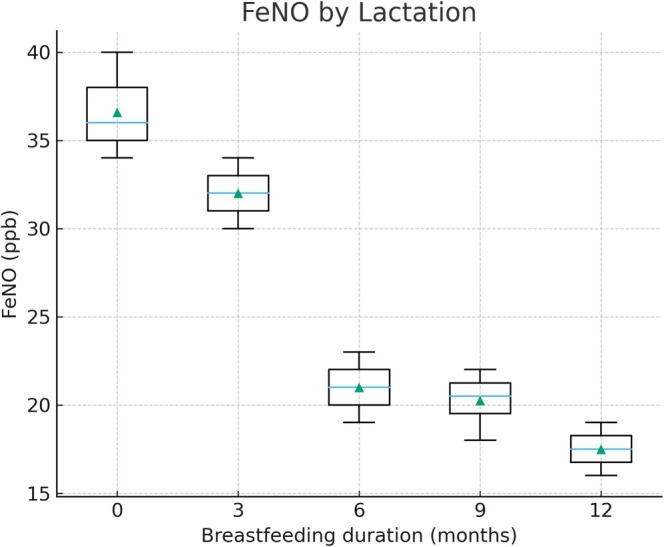
Fractional exhaled nitric oxide (FeNO) levels by breastfeeding duration. Boxplots represent FeNO levels (ppb) stratified by breastfeeding duration. Group sizes were < 6 months breastfeeding (*n* = 86) and ≥ 6 months breastfeeding (*n* = 76); between‐group differences were statistically significant (*p* < 0.01). Children breastfed for ≥ 6 months had significantly lower FeNO values compared with those with shorter or absent breastfeeding histories (*p* < 0.01). Boxes represent IQR, horizontal lines depict medians, and whiskers denote the 10th–90th percentiles.

Children breastfed for 6 months or longer demonstrated significantly higher ACT scores and lower FeNO values compared with those breastfed for shorter periods or not at all. The mean ACT score was 21.5 in the ≥ 6 months breastfeeding group versus 18.9 in the < 6 months group (*p* < 0.01), while median FeNO levels were 21 ppb versus 34 ppb, respectively (*p* < 0.01). Figure 1 illustrates the distribution of ACT scores by breastfeeding duration, and Figure 2 displays FeNO levels by breastfeeding duration.

**FIGURE 3:**
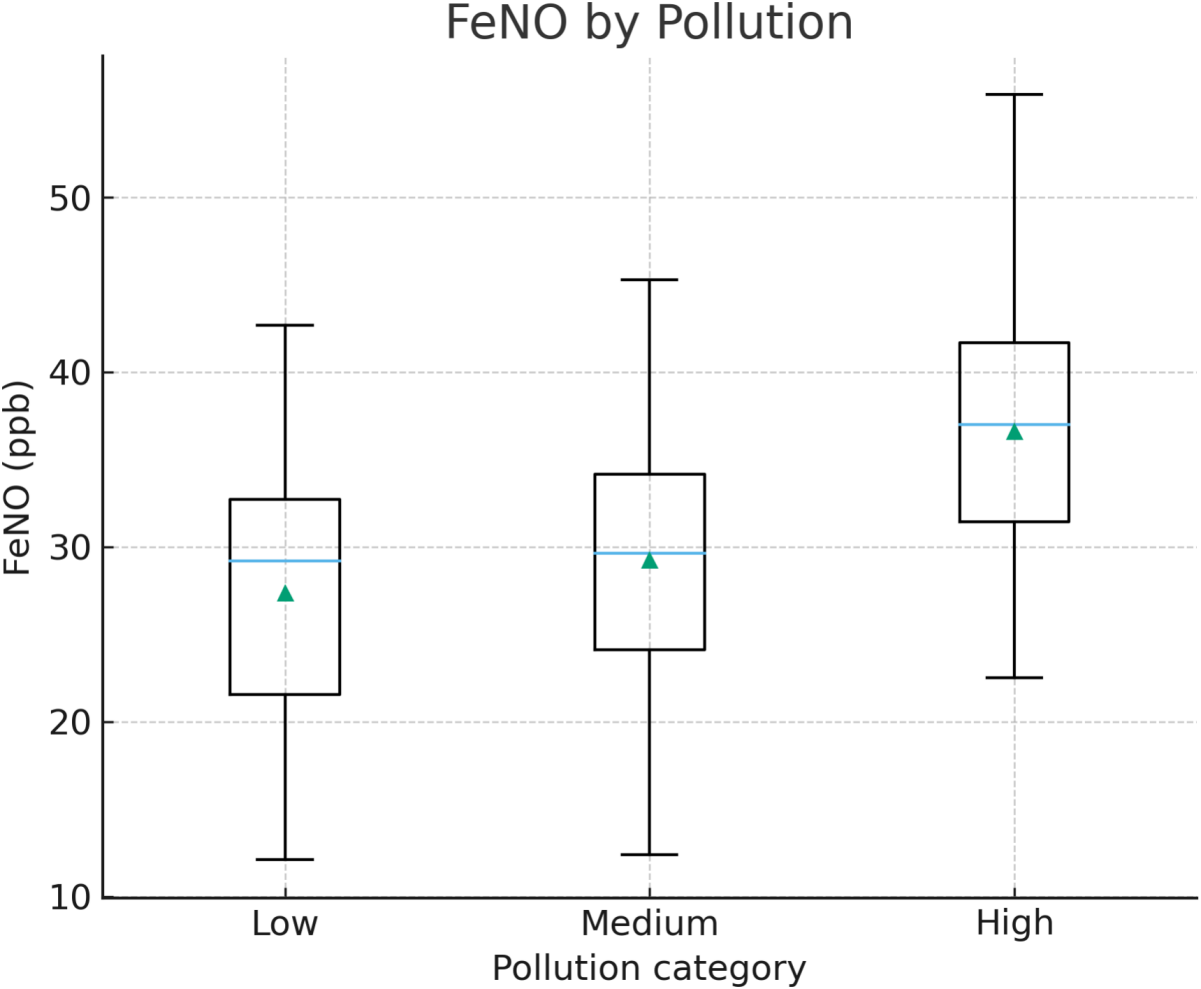
Fractional exhaled nitric oxide (FeNO) levels by pollution category. Boxplots show FeNO levels (ppb) among children residing in boroughs with high, medium, or low pollution categories according to historical concentrations. Group sizes were high pollution (*n* = 60), medium pollution (*n* = 67), and low pollution (*n* = 35); overall differences were statistically significant (*p* < 0.01). Children from high‐pollution boroughs presented significantly higher FeNO levels compared with those from low‐pollution areas (*p* < 0.01).

Pollution category was also associated with asthma outcomes. Children living in high‐pollution boroughs had lower mean ACT scores (18.7) and higher FeNO levels (median 36 ppb) compared with those in low‐pollution boroughs (mean ACT 21.8; median FeNO 23 ppb; *p* < 0.01). Figure 3 shows the distribution of FeNO levels according to pollution classification [17].

Multivariable regression models confirmed these associations. Breastfeeding for 6 months or longer was independently associated with adequate asthma control (ACT ≥ 20) with an adjusted odds ratio of 2.1 (95% CI 1.3–3.5, *p* < 0.01). In linear models, longer breastfeeding duration predicted significantly lower log‐transformed FeNO levels (β = −0.24, *p* < 0.01). High‐pollution exposure was independently associated with poorer asthma control and elevated FeNO, but interaction terms indicated that prolonged breastfeeding mitigated the adverse effects of pollution.

## 4 Discussion

This multicenter study provides new evidence that breastfeeding for at least 6 months is associated with clinically meaningful improvements in asthma control and airway inflammation, even in the context of high urban pollution, as measured by ACT scores and FeNO levels, respectively. Importantly, we also identified significant interactions between breastfeeding duration and borough‐level air pollution categories, suggesting that breastfeeding may partially mitigate the adverse respiratory impact of high environmental pollution [4, 28, 29]. These results expand the growing body of evidence linking early‐life nutrition and environmental exposures with asthma morbidity and align with recent international consensus statements advocating multidimensional assessment in pediatric asthma, incorporating both clinical outcomes and biomarkers [17, 29].

Our findings are consistent with multiple systematic reviews and meta‐analyses reporting that breastfeeding reduces the risk of asthma and allergic multimorbidity, particularly when extended beyond the first 6 months of life [3, 4, 15]. Unlike most prior studies that focused exclusively on prevalence or incidence, we examined two complementary outcomes: symptom control (ACT) and airway eosinophilic inflammation (FeNO). FeNO is a validated noninvasive biomarker, strongly associated with type‐2 inflammation and treatment responsiveness, and recent ATS/ERS statements reaffirm its role in pediatric asthma management [17, 18]. Our observation of significantly lower FeNO in children with longer breastfeeding duration suggests a biologically plausible mechanism: early immune modulation and mucosal protection that may dampen airway inflammation triggered by pollutants or allergens.

The interaction between breastfeeding and air pollution represents a novel and clinically relevant contribution. Mexico City, with its high burden of ozone and fine particulate matter, has been a natural laboratory for pediatric respiratory epidemiology. Numerous time‐series and cohort studies have linked pollutant exposure to increased asthma exacerbations and reduced lung function in children [10, 11, 23, 29, 30, 31, 32]. However, few studies have directly tested whether breastfeeding modifies this effect. This is the first study in Mexico City to demonstrate that breastfeeding duration modifies the impact of borough‐level pollution on both ACT and FeNO outcomes, reducing the susceptibility of children living in boroughs with high historical pollutants and high ozone averages. This finding supports earlier hypotheses from population‐based studies that breastfeeding can buffer environmental insults to the respiratory system.

Several mechanisms may explain this interaction. Human milk contains bioactive components—such as secretory IgA, cytokines, oligosaccharides, and polyunsaturated fatty acids—that contribute to mucosal defense, shape infant microbiota, and reduce oxidative stress [2, 4, 8, 13, 14, 28, 29]. In polluted environments, where airway epithelial injury and reactive oxygen species are elevated, breastfeeding may provide critical immunomodulatory support. The lower FeNO values observed among breastfed children in our cohort are consistent with this mechanistic framework.

The strengths of our study include its multicenter design across diverse boroughs of Mexico City, the use of both subjective and objective asthma outcome measures, and the integration of borough‐level pollution data obtained from official monitoring reports [7, 21]. Additionally, our use of multivariable regression with interaction terms allows for a more nuanced understanding of how breastfeeding and pollution jointly influence asthma outcomes. These features enhance both the internal and external validity of our findings.

Several limitations should be acknowledged. The retrospective design introduces potential recall bias in parental reporting of breastfeeding duration, which lacks granularity regarding exclusivity and daily feeding frequency. Additionally, allergen sensitization status was not systematically available for all participants, limiting adjustment for allergic profiles that may influence FeNO levels. Pollution exposure was classified at the borough level and may not fully capture individual variability. Finally, although the sample size was adequate for primary analyses, residual confounding cannot be excluded. These limitations underscore the need for prospective studies with standardized allergy testing, detailed breastfeeding characterization, and individual‐level exposure assessment.

Despite these limitations, our study provides valuable insight into the interplay between early‐life nutrition and environmental exposures in pediatric asthma. The public health implications are considerable: Breastfeeding promotion should be prioritized as a modifiable protective factor, particularly for children in high‐pollution urban settings [2, 7, 21]. At the clinical level, the incorporation of FeNO into routine care may help identify subgroups of children with ongoing type‐2 inflammation, guiding step‐up therapy or referral to specialized care, in line with current GINA and ATS/ERS recommendations [1, 5, 17, 33].

Future research should move toward prospective, longitudinal designs with repeated ACT and FeNO measurements, integrating personal exposure monitoring (e.g., portable sensors for NO_2_, O_3_, and PM_2.5_) and including allergen sensitization profiling [7, 29, 30, 31]. Large multicenter studies across different cities with varying pollution burdens are needed to confirm whether the protective role of breastfeeding is generalizable. Additionally, interventional research on FeNO‐guided management strategies in high‐pollution pediatric settings would help translate these findings into practice.

## 5 Conclusion

Breastfeeding for at least 6 months is associated with better asthma control and lower airway inflammation in children, even in the context of high urban pollution. Promoting breastfeeding and integrating biomarker‐guided care with environmental health policies can meaningfully improve pediatric asthma outcomes in megacities.

These findings highlight breastfeeding as an actionable and low‐cost strategy to improve pediatric asthma outcomes, particularly in highly polluted urban environments. Integrating breastfeeding promotion with biomarker‐guided care and urban health policies may substantially reduce the long‐term respiratory burden of pediatric asthma in polluted megacities.

## Author Contributions

Conceptualization: Víctor González‐Uribe and Zaira Selene Mojica‐González. Methodology: Víctor González‐Uribe, Zaira Selene Mojica‐González, and María Julia Rendón‐Salazar. Data curation: Jimena Prieto‐Gómez, Luis Ángel Hernández‐Zárate, and Ricardo Martinez‐Tenopala. Formal analysis: Víctor González‐Uribe and Ricardo Martinez‐Tenopala. Writing – original draft: Víctor González‐Uribe and Zaira Selene Mojica‐González. Writing – review and editing: all authors. Supervision: Víctor González‐Uribe.

## Funding

The authors have nothing to report.

## Ethics Statement

The protocol was reviewed and approved by the institutional ethics committees of all participating centers. Given the retrospective design, informed consent was waived. Patient confidentiality was safeguarded throughout.

## Conflicts of Interest

The authors declare no conflicts of interest.

## Data Availability

The data that support the findings of this study are available on request from the corresponding author. The data are not publicly available due to privacy or ethical restrictions.
